# Bioinformatics-integrated screening of systemic sclerosis-specific expressed markers to identify therapeutic targets

**DOI:** 10.3389/fimmu.2023.1125183

**Published:** 2023-03-30

**Authors:** Jiahui Jin, Yifan Liu, Qinyu Tang, Xin Yan, Miao Jiang, Xu Zhao, Jie Chen, Caixia Jin, Qingjian Ou, Jingjun Zhao

**Affiliations:** ^1^ Department of Dermatology, Tongji Hospital, School of Medicine, Tongji University, Shanghai, China; ^2^ Department of Dermatology, Xinhua Hospital, School of Medicine, Shanghai Jiaotong University, Shanghai, China

**Keywords:** systemic sclerosis, tissue-specific expressed genes, biomarkers, RNA regulatory pathway, drug-gene interaction

## Abstract

**Background:**

Systemic sclerosis (SSc) is a rare autoimmune disease characterized by extensive skin fibrosis. There are no effective treatments due to the severity, multiorgan presentation, and variable outcomes of the disease. Here, integrated bioinformatics was employed to discover tissue-specific expressed hub genes associated with SSc, determine potential competing endogenous RNAs (ceRNA) regulatory networks, and identify potential targeted drugs.

**Methods:**

In this study, four datasets of SSc were acquired. To identify the genes specific to tissues or organs, the BioGPS web database was used. For differentially expressed genes (DEGs), functional and enrichment analyses were carried out, and hub genes were screened and shown in a network of protein-protein interactions (PPI). The potential lncRNA–miRNA–mRNA ceRNA network was constructed using the online databases. The specifically expressed hub genes and ceRNA network were validated in the SSc mouse and in normal mice. We also used the receiver operating characteristic (ROC) curve to determine the diagnostic values of effective biomarkers in SSc. Finally, the Drug-Gene Interaction Database (DGIdb) identified specific medicines linked to hub genes.

**Results:**

The pooled datasets identified a total of 254 DEGs. The tissue/organ-specifically expressed genes involved in this analysis are commonly found in the hematologic/immune system and bone/muscle tissue. The enrichment analysis of DEGs revealed the significant terms such as regulation of actin cytoskeleton, immune-related processes, the VEGF signaling pathway, and metabolism. Cytoscape identified six gene cluster modules and 23 hub genes. And 4 hub genes were identified, including Serpine1, CCL2, IL6, and ISG15. Consistently, the expression of Serpine1, CCL2, IL6, and ISG15 was significantly higher in the SSc mouse model than in normal mice. Eventually, we found that MALAT1-miR-206-CCL2, let-7a-5p-IL6, and miR-196a-5p-SERPINE1 may be promising RNA regulatory pathways in SSc. Besides, ten potential therapeutic drugs associated with the hub gene were identified.

**Conclusions:**

This study revealed tissue-specific expressed genes, SERPINE1, CCL2, IL6, and ISG15, as effective biomarkers and provided new insight into the mechanisms of SSc. Potential RNA regulatory pathways, including MALAT1-miR-206-CCL2, let-7a-5p-IL6, and miR-196a-5p-SERPINE1, contribute to our knowledge of SSc. Furthermore, the analysis of drug-hub gene interactions predicted TIPLASININ, CARLUMAB and BINDARIT as candidate drugs for SSc.

## 1 Introduction

Systemic sclerosis (SSc) is a chronic autoimmune disease characterized by vasculopathy, immune dysregulation, and progressive fibrosis of the skin and internal organs. Fibrosis might be viewed as a maladaptive repair procedure (1, 2). Normal tissue architecture was replaced by connective tissue, which was rich in collagen and other extracellular matrix (ECM) molecules. Since there is no effective therapy, SSc frequently results in severe impairment or even death. Therefore, in-depth research on the pathogenesis of and potential treatments for SSc is the focal point of prevalent studies.

Bioinformatics analysis of published transcriptomic datasets have been extensively applied to investigate diseases’ pathogenesis and are being utilized increasingly to find novel and effective biomarkers for diseases (3). The competitive endogenous RNA (ceRNA) theory revealed that a transcriptional regulatory network contributes to disease etiology. It is reported that ceRNAs are functional regulatory molecules that moderate essential physiological procedures, including mRNA stability, chromosomal modifications, and translation, as well as important signaling pathways (4, 5). MicroRNAs (miRNAs) and long non-coding RNAs (lncRNAs) may be underlying regulators and contribute to the fibrotic responses of SSc (6, 7). MiR-142-3p serum levels may be greatly elevated in SSc patients, corresponding with the severity of the condition (8). MiRNA-29b levels were lower in SSc patients’ skin tissues and correlated with collagen expression (9–12). Mai A Abd-Elmawla et al. investigated the lncRNA expression profiles in SSc patients and purposed four lncRNAs that may be new and effective biomarkers for SSc, including SPRY4-IT1, HOTTIP, ANCR and TINCR. SPRY4-IT1 might contribute to the diagnosis and differentiate of SSc patients between subtypes (13).

Circular RNAs (circRNAs) are a distinct type of RNA molecule formed by the covalent linking of linear RNA by back-splicing. CircRNAs act as ceRNAs, and they can modify the activity of miRNAs by attaching to miRNA molecules and limiting their availability to bind to and suppress protein coding transcripts. CircRNAs have multiple regulatory effects on fibroblast activation and the overproduction of fibrosis-associated proteins (14). The downregulation of CircACTR2 greatly reduced collagen IV and fibronectin. It plays promoting functions in the onset of renal fibrosis (15). In addition, downregulation of circHIPK3 can reduce cardiac fibrosis post-myocardial infarction in mice (16). The understanding of the molecular processes of the ceRNA regulator network is anticipated to lead to improvements in SSc diagnosis and treatment innovations.

In our study, we predicted the ceRNA of screened hub genes and constructed the ceRNA networks from the published datasets of SSc. With the use of the Drug-Gene Interaction Database (DGIdb), hub gene-related prospective and efficient medicines were identified. Our research explores effective biomarkers for SSc and sheds light on the transcriptome-level etiology. It also provides some theoretical support for investigating potential drugs for the therapy of SSc.

## 2 Method

### 2.1 Datasets acquisition

The datasets of skin samples involved in this study were downloaded from the GEO database (http://www.ncbi.nlm.nih.gov/geo). The inclusion requirements were as follows: (1) Homo sapiens gene expression profiling, including RNA-seq datasets and microarray datasets; (2) skin tissue from SSc patients; (3) datasets containing more than six skin specimens; and (4) each subject’s biopsy sample was not duplicated. Ultimately, GSE95065, GSE125362 (17), and GSE32413 (18, 19) were assigned as test sets, and the GSE130955 (20, 21) dataset was selected as the validation set. **Table 1** displays the datasets’ specifics.

**Table 1:** The details of selected datasets.

GEO	Platform	Samples No		Source tissue	Experiment type	Attribute
		Normal	SSc			
GSE95065	Affymetrix Human Genome U133A 2.0 Array	11	18	Skin	Array	Test
GSE125362	Agilent-014850 Whole Human Genome Microarray 4x44K G4112F	3	7	Skin	Array	Test
GSE32413	Agilent-014850 Whole Human Genome Microarray 4x44K G4112F	7	9	Skin	Array	Test
GSE130955	Illumina HiSeq 2500 (Homo sapiens)	24	31	Skin	RNA-Seq	Validation

### 2.2 Data preprocessing and screening of DEGs

Three test microarray datasets, GSE95065, GSE125362, and GSE32413, were obtained for subsequent analysis. GES32413 and GSE130955 provide clinical data, including age, sex, disease duration, the modified Rodnan skin score (MRSS), forced vital capacity (FVC), and so on (**Supplementary Tables 5, 6**). After merging three gene matrices, the heterogeneity resulting from multiple experimental batches and platforms was eliminated using the R program “sva”. The merging gene expression matrix was examined using the limma package. Next, the gene expression matrix of GSE130955 was downloaded and examined using the limma package. DEGs have to meet the screening requirements of |log fold change (log FC)| > 1 and an adjusted p value of 0.05. The p value was adjusted using the false discovery rate (FDR).

### 2.3 Establishment of the SSc mouse model

Animal studies were conducted in accordance with the recommendations of the Institute for Laboratory Animal Resources at Tongji University. To generate the SSc model, subcutaneous injections of bleomycin (BLM, R25001, Thermo Fisher Scientific, USA) solution were used (22). In detail, 100 μL of BLM PBS solution (1 mg/mL) was subcutaneously injected into the shaved backs (1 cm^2^) of mice once a day for 28 consecutive days. Mice in the normal group received 100 μL of PBS. Finally, mice were sacrificed, and skin biopsies were obtained. Samples were fixed in 4% paraformaldehyde (E672002, Sangon Biotech, China) for histology or stored at -80 °C for molecular analyses.

### 2.4 RNA extraction and Quantitative real-time PCR

Skin samples were treated with the TRIzol reagent (9109, Takara, Japan). Then, total RNA was extracted in accordance with the manufacturer’s instructions. Next, RNA was reverse transcribed into cDNA using PrimeScript™ RT Master Mix (RR036A, Takara, Tokyo). qRT-PCR was carried out using the specific primers (**Supplementary Table 1**) and SYBR green qPCR master mix (FP205-03, Tiangen, China). In each sample, the average level of GAPDH mRNA was used to normalize gene expression. The 2^ΔΔCt^ method was utilized to calculate the fold change in expression.

### 2.5 Western blot

Skin samples were collected and lysed with RIPA buffer (C500005, Sangon Biotech, China) containing protease and phosphatase inhibitors (C0001 and C0004, TargetMol, USA). Next, SDS-PAGE (C671102, Sangon Biotech, China) was applied to separate the protein, which was then blotted to PVDF membranes (IPFL85R, Merck, Germany). After incubation with the primary antibodies (**Supplementary Table 2**), the PVDF membranes were incubated with HRP-conjugated secondary antibodies. Finally, the protein bands were examined using the ECL western blotting substrate (E411, Vazyme, China) and a Tanon chemiluminescence image detection system (5200S, Tanon, China) (23).

### 2.6 Immunohistochemical analysis

The skin tissues were fixed at room temperature with 4% paraformaldehyde and embedded in paraffin to create paraffin sections (10 μm). Deparaffinized paraffin-embedded skin tissue sections were treated with primary antibodies (**Supplementary Table 2**) for immunohistochemistry examination. Sections were incubated HRP-labeled secondary. And then, the mouse tissue sections were incubated with diaminobenzidine (DAB) (E670033, Sangon Biotech, China) solution for 1 minute.

### 2.7 Authentication of tissue/organ−specific expressed genes

The database BioGPS (http://biogps.org/) was utilized to examine the tissue/organ-specific expression of DEGs (24). The evaluation standards were followed: (1) the expression of transcripts corresponding to a single organ system was more than ten times higher than the median, and (2) expression in the second-most abundant tissue was only one-third as high (25). These criteria led to the conclusion that the DEGs identified were tissue/organ specific.

### 2.8 Functional enrichment analysis

Enrichment analyses for DEGs were performed using the R tool “clusterProfiler”. The threshold p value < 0.05 was used to identify the significantly different data. Metascape (http://metascape.org) contributes to gene annotation and analysis that identifies common and distinctive pathways across a set of disparate target-discovery investigations. Next, Metascape was applied to assess the KEGG pathway for DEGs.

### 2.9 Construction of the PPI network and identification of hub genes

STRING (http://string-db.org/) is software that can assess and build networks between proteins (26). With a cut-off condition (combined score > 0.4), STRING was utilized to assess the integrated interaction of DEGs at the protein level. Then, Cytoscape (27) (https://cytoscape.org/) is used to create the PPI network and produce the crucial gene clusters. The top 8 algorithms (MCC, EPC, EcCentricity, Degree, MNC, Stress, Closeness, and Radiality) were performed to recognize the hub genes. The “UpSet” R package was applied to identify hub genes in the top 50 node genes for each method.

### 2.10 Evaluation of the diagnostic significance of the hub genes

Receiver Operating Characteristic (ROC) curve analysis was applied to assess the potential and effective biomarkers in GSE95065, GSE125362, GSE32413 and GSE130955. Moreover, the predictive value of biomarkers was identified in accordance with the area under the ROC curve (AUC) value.

### 2.11 Construction of the miRNA-mRNA network

To understand the relationship between messenger RNA (mRNA) and miRNA, five online miRNA databases, including Tarbase, miRWalk, DIANA-microT, Targetscan, and miRDB, were applied to predict target miRNAs. In addition, at least three datasets included the target miRNAs. Next, the miRNA-mRNA network was established by Cytoscape for better visualization.

### 2.12 ceRNA networks construction

StarBase (http://starbase.sysu.edu.cn/index.php) has been described for decoding the interaction networks of miRNAs, lncRNAs, and mRNAs from CLIP-Seq data, RNA-binding proteins (RBPs), and competing endogenous RNAs (ceRNAs), and it has also been developed for miRNA-target interactions, such as miRNA-lncRNA, miRNA-mRNA, miRNA-circRNA, and so on (28). Using Starbase, we predicted lncRNAs and circRNAs according to the target miRNAs. Finally, CeRNA networks were constructed using Cytoscape.

### 2.13 *In situ* hybridization

To exactly localize the expression of miR-196a-5p, miR-206, Let-7a-5p, LncRNA MALAT, and LncRNA Xist in mouse skin tissue, probes labeled by 3’-tailing with digoxin were synthesized by Sangon Biotech (Shanghai, China) (**Supplementary Table 3**). The enhanced-sensitivity *in situ* hybridization (ISH) detection kit (Bosterbio, Wuhan, China) was applied to carry out lncRNA and miRNA ISH. According to the preceding description, the sections of skin tissue embedded in paraffin were deparaffinized (29) and digested in 3% citric acid diluted pepsin for 30 min at 37°C. The sections were treated with digoxin labeled probes diluted by hybridization diluent overnight at 37°C. After hybridization, the skin tissue sections were washed three times with saline sodium citrate (SSC; Bosterbio, Wuhan, China) for 5 min and then blocked in blocking solution for 30 min at 37°C. Next, the alkaline phosphatase-labeled mouse anti-digoxigenin was used to incubate the sections for 1 h at 37°C. 5-Bromo-4-Chloro-3-Indolyl Phosphate (BCIP)/Nitro-Blue-Tetrazolium (NBT) was applied at 37°C for 60 min. Finally, sections were sealed with a water-soluble seal.

### 2.14 Dual-luciferase reporter gene assay

The wild-type (WT) or mutant (MUT) 3’-UTR fragments of IL-6, CCL2, and LncRNA MALAT were respectively cloned into the psiCHECK-2 dual luciferase miRNA target expression vector. The HEK293T cells were seeded in 48-well plates to reach approximately 80% confluency at the time of the transfection. The WT or MUT plasmids (200 ng/well) were co-transfected with miRNA mimics (100 nM) or control mimics (mimic-NC, 100 nM) into HEK293T cells using lipofectamine 3000 transfection reagent (L3000001, Thermo Fisher Scientific, USA). At 48 h, the Renilla and Frefy luciferase activities were conducted using a dual luciferase reporter gene assay kit (11402ES60, Yeasen, China) according to the manufacturer’s instructions.

### 2.15 Construction of the drugs-genes network

The DGIdb (https://dgidb.genome.wustl.edu) is a database that allows you to search lists of genes against existing databases of known or prospective drug-gene interactions (30). We searched the DGIDB for candidate drugs linked to these hub genes.

### 2.16 Statistical analysis

R software was applied to conduct the statistical analyses. For the animal experiments, the variations between the two groups were examined by a student’s t-test with GraphPad Prism 9.5.1 (GraphPad Software, USA). P-value < 0.05 was considered statistically significant.

## 3 Result

### 3.1 Identification of significant DEGs

**Figure 1:**
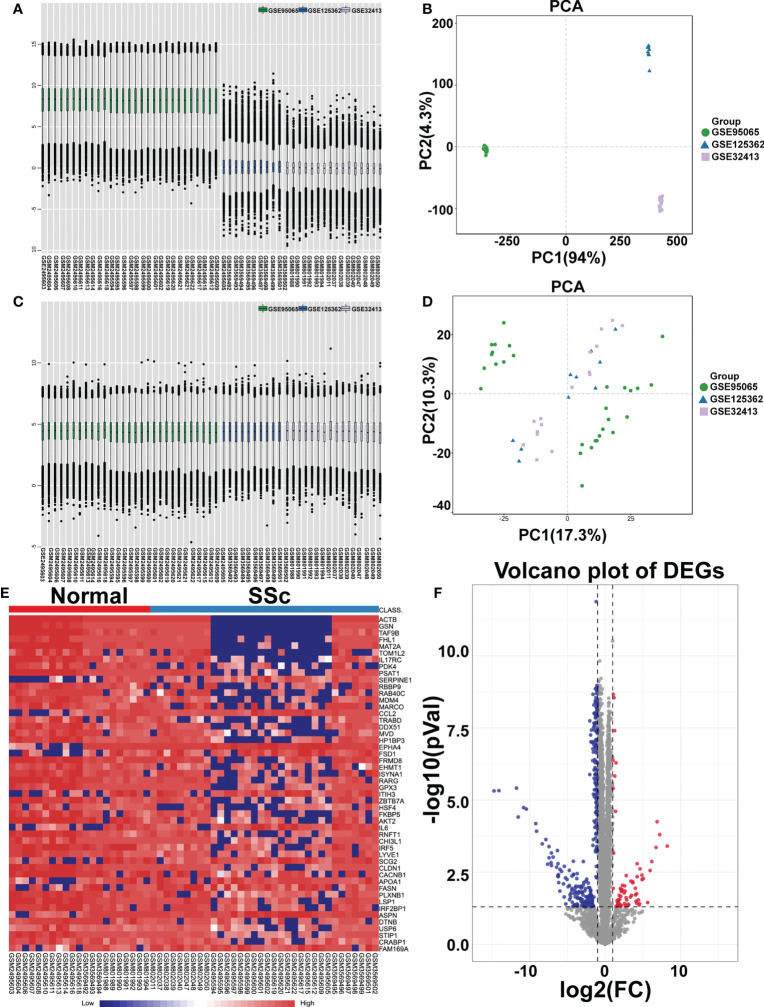
Identification of differentially expressed mRNAs (DEGs). Box plot and principal component analyses were applied to remove batch correction of GSE95065, GSE125362, and GSE32413. **(A, B)** Before batch correction and **(C, D)** After batch correction. **(E)** Heatmap of DEGs between the SSc group and the normal group (red represents high expression; blue represents low expression). **(F)** Volcano plot of DEGs between the SSc group and the normal group. The red plots represent upregulated genes, the blue plots represent downregulated genes, the black plots represent nonsignificant genes.

To analyze the DEGs, the datasets GSE95065, GSE125362, and GSE32413 were screened, including 21 normal samples and 34 SSc samples. The data before (**Figures 1A, B**) and after (**Figures 1C, D**) batch correction is shown in **Figure 1**, demonstrating the successful elimination of the batch impact of the combined data. Totally, 254 DEGs were discovered, including 214 downregulated genes and 40 upregulated genes, in the SSc samples when compared to the genes in the normal samples (**Supplementary Table 4**). As shown in **Figures 1E, F**, the heatmap and volcanic map displayed the final DEGs. We re-analyzed GSE130955 dataset and the results obtained using both datasets are presented side by side for comparison. Total 367 DEGs were discovered, including 327 upregulated genes and 40 downregulated genes, in the SSc samples when compared to the genes in the normal samples (**Supplementary Figure 1**).

### 3.2 Identification of the tissue/organ−specific expressed genes

We continued to use BioGPS to screen 72 tissue/organ-specific expressed genes. These genes were found to be particularly showed in the haematologic/immune system (41.67%) and bone/muscle tissue (12.50%), as shown in **Table 2**. The nervous system was followed by the placenta system (1.39%), the digestive system (6.94%), the endocrine system (4.17%), the genital system (6.94%), the respiratory system (2.78%), the circulatory system (1.39%), and the other (8.33%).

**Table 2:** Distribution of tissue/organ-specifically expressed genes identified by BioGPS.

System/Organ	Genes	Counts
Haematologic/Immune	PSAT1, TRABD, LSP1, STIP1, C20orf27, CLEC2D, HNRNPH1, S100A11, ISG15, DEF6, DGKA, POLR2E, EMP3, WBP11, PLEKHA1, CCNL2, NADK, MFHAS1, FXYD5, TAGLN2, C1orf54, CNN2, ST6GALNAC4, GSR, BCL2L1, DDX3X, NBEAL2, TAP1, SELENBP1, FLI1	30
Bone/muscle	SERPINE1, CCL2, IL6, STC2, RCN3, PRSS23, OSMR, TRIP10, MFAP2	9
Nervous	FSD1, SCG2, FAM169A, REEP2, CDH10, SCRG1, STMN2, CHN1	8
Placenta	FBLN1	1
Digestive	ITIH3, APOA1, APOE, SHMT1, DCXR	5
Endocrine	CRABP1, TM7SF2, PDK4	3
Genital	ASPN, GAS1, UTRN, OSR2, ISYNA1	5
Respiratory	MFAP4, MARCO	2
Circulatory	UQCRC1	1
Other		
Adipose	FKBP5, FASN, LPL, FBLN5	4
Tongue	IL1RN	1
trachea	ALDH3A1	1

### 3.3 Enrichment analyses of DEGs of SSc

**Figure 2:**
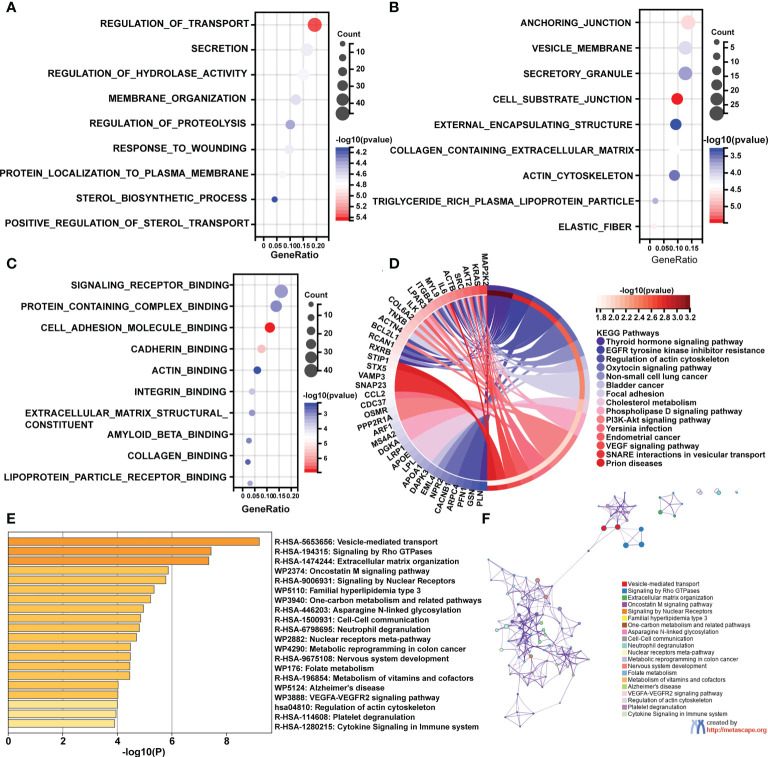
GO and KEGG pathway enrichment analyses of differentially expressed mRNAs (DEGs). **(A)** The bubble plot shows the top 10 enriched biological processes of DEGs. **(B)** The bubble plot shows the top 10 enriched cellular components of DEGs. **(C)** The bubble plot shows the top 10 enriched molecular functions of DEGs. **(D)** The chord plot shows the most enriched KEGG pathways of DEGs. **(E, F)** The KEGG pathway enrichment analysis of DEGs using Metascape.

Following that, enrichment studies were carried out using the clusterProfiler package and Metascape. To determine the enrichment pathway of DEGs, we examined them from three different perspectives, including biological processes (BP), cellular components (CC), molecular functions (MF), and KEGG pathways. The biological processes analysis concentrates on regulation of transport, membrane organization, regulation of proteolysis, response to wounding, protein localization to plasma membrane, sterol biosynthetic process, and positive regulation of sterol transport (**Figure 2A**). The main cellular components of DEGs contained cell substrate junction, elastic fiber, collagen containing extracellular matrix, and actin cytoskeleton (**Figure 2B**). The main molecular functions of DEGs are involved in signaling receptor binding, actin binding, extracellular matrix structural constituents, and collagen binding (**Figure 2C**). As shown in **Figure 2D**, the top 15 KEGG pathways were screened and visualized by chord plot. DEGs were abundant in the actin cytoskeleton regulation, the oxytocin signaling pathway, cholesterol metabolism, the PI3K-Akt signaling pathway, and the VEGF signaling pathway. In addition, the DEGs were also significantly related to immune-related processes, including neutrophil degranulation and cytokine signaling in the immune system (**Figures 2E, F**).

To confirm this analysis, we further performed GO and KEGG enrichment analyses from GSE130955. The biological processes analysis concentrates on immune effector process, secretion, external encapsulating structure organization, wounding healing, and regulation of complement activation (**Supplementary Figure 2A**). The main cellular components of DEGs contained collagen containing extracellular matrix, collagen trimer, elastic fiber and actin filament (**Supplementary Figure 2B**). The main molecular functions of DEGs are involved in signaling receptor binding, extracellular matrix structural constituents, collagen binding, and extracellular matrix binding (**Supplementary Figure 2C**). As shown in **Supplementary Figure 2D**, the top 15 KEGG pathways were screened and visualized by chord plot. DEGs were abundant in the ECM-receptor interaction, focal adhesion, cytokine-cytokine receptor interaction, PI3K-Akt signaling pathway, Arachidonic acid metabolism, IL-17 signaling pathway, and TNF signaling pathway. Therefore, the analyses of GSE130955 are consistent with the merged array data, and confirm the common functions of DEGs of SSc.

### 3.4 Construction of PPI network and identification of hub gene

**Figure 3:**
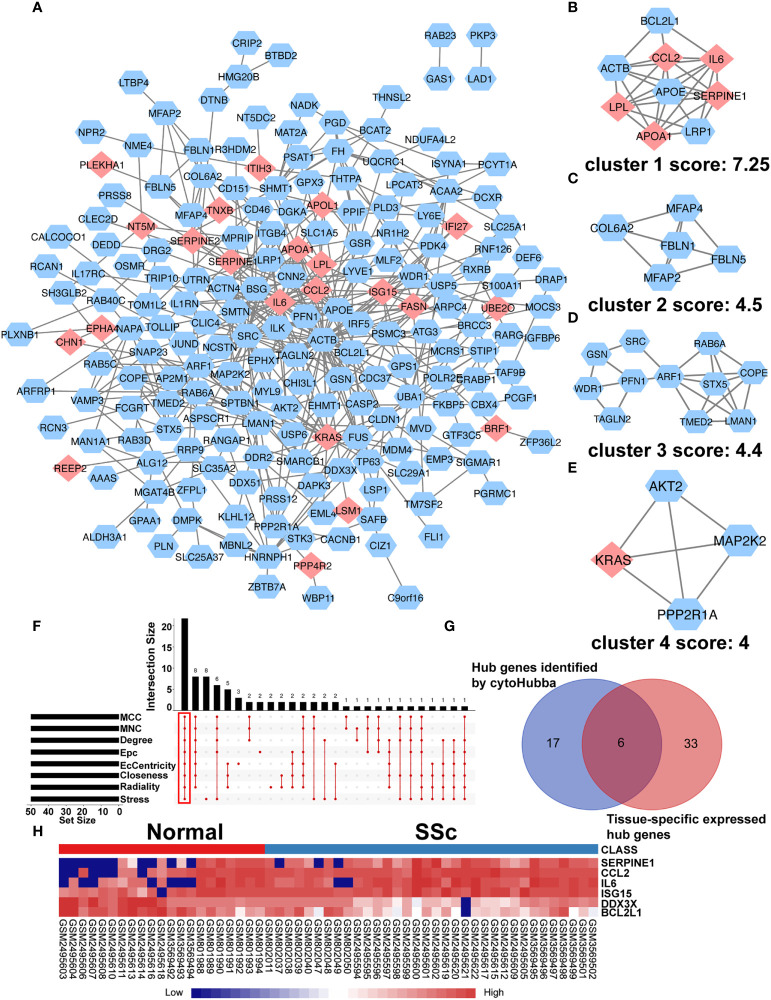
PPI network of DEGs and Identification of Hub Genes. **(A)** The interaction network between proteins was constructed using cytoscape. Each node represents a protein, while each edge represents one protein–protein connection. Pink diamonds represent the upregulated genes, and blue octagons represent the downregulated genes. Cluster modules are extracted by MCODE. Cluster 1 **(B)** received the best cluster score (score: 7.25, 9 nodes and 27 edges), whereas Cluster 2 **(C)** came in second place (score: 4.5, 5 nodes and 9 edges), Cluster 3 **(D)** came in third (score: 4, 11 nodes and 23 edges), and Cluster 4 **(E)** came in fourth (score: 4, 4 nodes and 6 edges). **(F)** Eight algorithms to identify hub genes by R package “UpSet”. **(G)** A Venn diagram was used to identify the six tissue-specific expressed hub genes in SSc. **(H)** The heatmap showed the expression of six tissue-specific expressed hub genes using merged microarray data.

To further understand interactions between DEGs, the PPI network was constructed in STRING (**Supplementary Figure 3**; **Figure 3A**). As shown in **Figures 3B-E**, Cluster 1 received the best cluster score (score: 7.25, 9 nodes and 27 edges), whereas Cluster 2 came in second place (score: 4.5, 5 nodes and 9 edges), Cluster 3 came in third (score: 4, 11 nodes and 23 edges), and Cluster 4 came in fourth (score: 4, 4 nodes and 6 edges). Moreover, 23 hub genes were discovered by combining the output of cytohubba’s eight algorithms—MCC, MNC, EPC, Degree, EcCentricity, Radiality, Closeness, and Stress [16] (**Figure 3F**). **Table 3** showed the extensive information of above hub genes. Additionally, according to BioGPS, the majority of DEGs were specifically expressed in the haematologic/immune and bone/muscle systems (54.17%).

**Table 3:** Hub genes are identified by 8 algorithms of cytoHubba.

Gene symbol	Description	log2FC	p value	Regulation
IL6	interleukin 6	5.9539	0.0027561	Up
APOE	apolipoprotein E	-3.0803	0.015316	Down
SERPINE1	serpin family E member 1	8.2288	0.00039285	Up
CCL2	C-C motif chemokine ligand 2	7.2248	0.00015671	Up
APOA1	apolipoprotein A1	4.8895	0.0057118	Up
SRC	SRC proto-oncogene, non-receptor tyrosine kinase	-3.9153	0.010908	Down
KRAS	proto-oncogene, GTPase	1.0537	0.0000039327	Up
BCL2L1	BCL2 like 1	-1.1615	0.000025821	Down
ARF1	ADP ribosylation factor 1	-1.3133	0.000000004148	Down
PFN1	profilin 1	-1.6611	0.000000096255	Down
WDR1	WD repeat domain 1	-1.13	0.000000002041	Down
MAP2K2	mitogen-activated protein kinase kinase 2	-1.7481	0.000000098617	Down
PPP2R1A	protein phosphatase 2 scaffold subunit Aalpha	-1.9288	0.000000055547	Down
GSN	gelsolin	-14.058	0.0000047037	Down
RAB6A	member RAS oncogene family	-2.8201	0.0071318	Down
ISG15	SG15 ubiquitin like modifier	2.1472	0.014642	Up
ITGB4	ntegrin subunit beta 4	-1.893	0.013537	Down
FASN	fatty acid synthase	4.865	0.0039771	Up
FUS	FUS RNA binding protein	-1.0197	0.000000004408	Down
UTRN	utrophin	-1.1604	0.000025949	Down
SMARCB1	Related, matrix associated, actin dependent regulator of chromatin, subfamily b, member 1	-1.0434	0.000000033976	Down
DDX3X	DEAD-box helicase 3 X-linked	-1.0819	0.000014644	Down
ACTB	actin beta	-14.71	0.0000048143	Down

To better comprehend the level of these hub genes in different tissues and organs, 23 hub genes were intersected with genes particularly expressed in the haematologic/immune and bone/muscle systems. Finally, six hub genes were identified and displayed in a heatmap, including SERPINE1, CCL2, IL6, ISG15, DDX3X, and BCL2L1 (**Figures 3G, H**). Interestingly, SERPINE1, CCL2, IL6, and ISG15 expression were increased drastically in SSc samples (P < 0.05) (**Supplementary Figures 4A-D**). DDX3X and BCL2L1 expression in SSc samples was lower than in the normal control (P < 0.05) (**Supplementary Figures 4E, F**). Thus, SERPINE1, CCL2, IL6, and ISG15 may be effective biomarkers for SSc.

### 3.5 Verification of the prospective biomarkers in SSc mouse model

**Figure 4:**
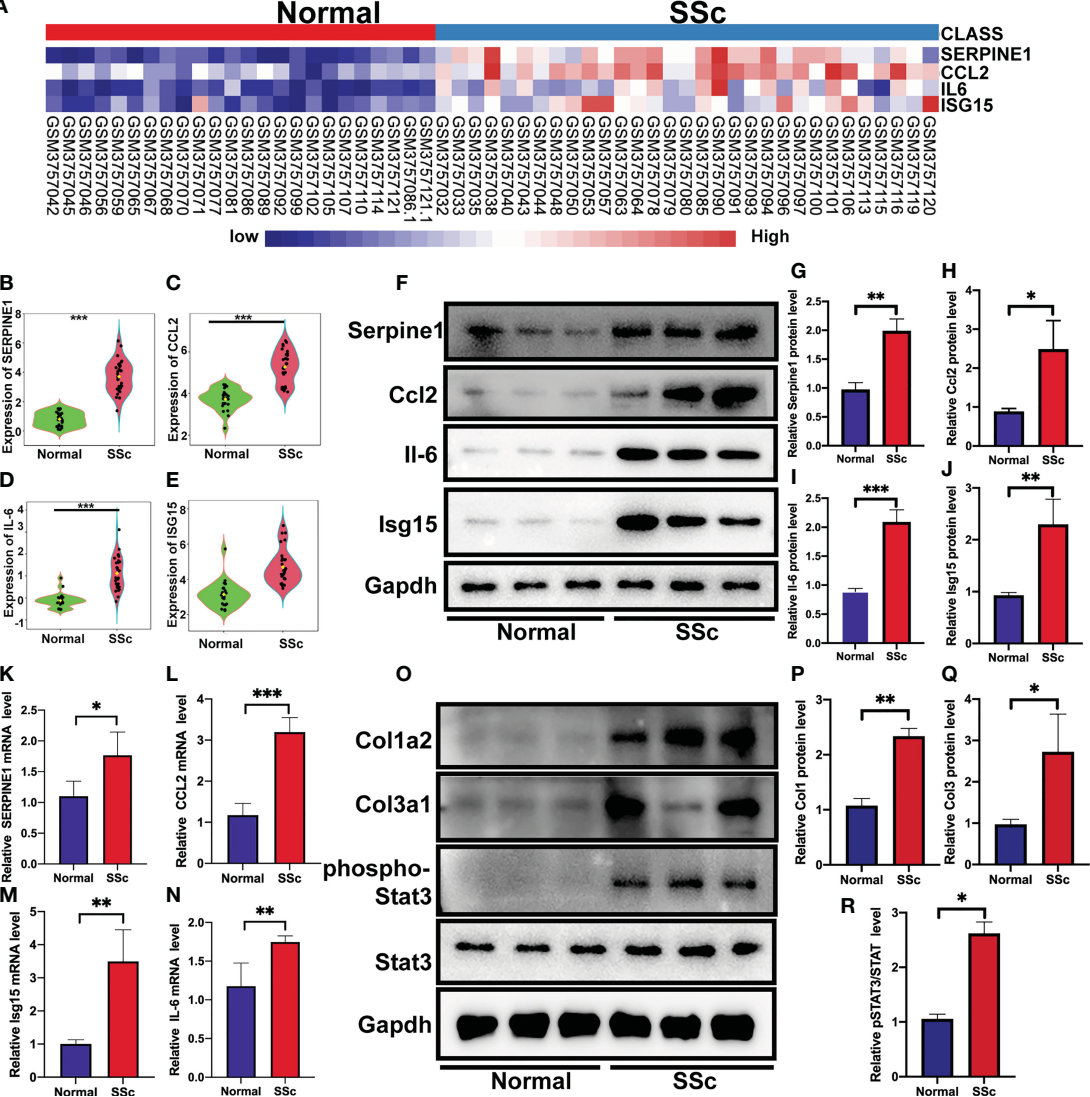
Verification of the 4 specifically expressed hub genes. **(A)** The heatmap showed the expression of four tissue-specifically expressed hub genes using the validation dataset. **(B-E)** The violin plot showed the detailed expression of four tissue-specific expressed hub genes using validation dataset. **(F-J)** Western blot showed the protein expression of Serpine1, Ccl2, Isg15 and Il6 in mouse skin tissue. **(K-N)** Quantification of Serpine1, Ccl2, Isg15 and Il6 mRNA in mouse skin tissue. **(O–R)** Western blot showed the protein expression of Col1a2, Col3a1, phospho-STAT3, and STAT3 in mouse skin tissue. *P < 0.05; **P < 0.01; ***P < 0.001.

Following that, the RNA-Seq dataset GSE130955, which included 24 normal samples and 31 SSc samples, was examined to validate the above analysis of four hub genes, including SERPINE1, CCL2, IL6, and ISG15. We depicted the expression of SERPINE1, CCL2, IL6, and ISG15 in a heatmap (**Figure 4A**). The level of the four hub genes in SSc samples was higher than in normal samples, which is consistent with the aforementioned findings (**Figures 4B-E**).

**Figure 5:**
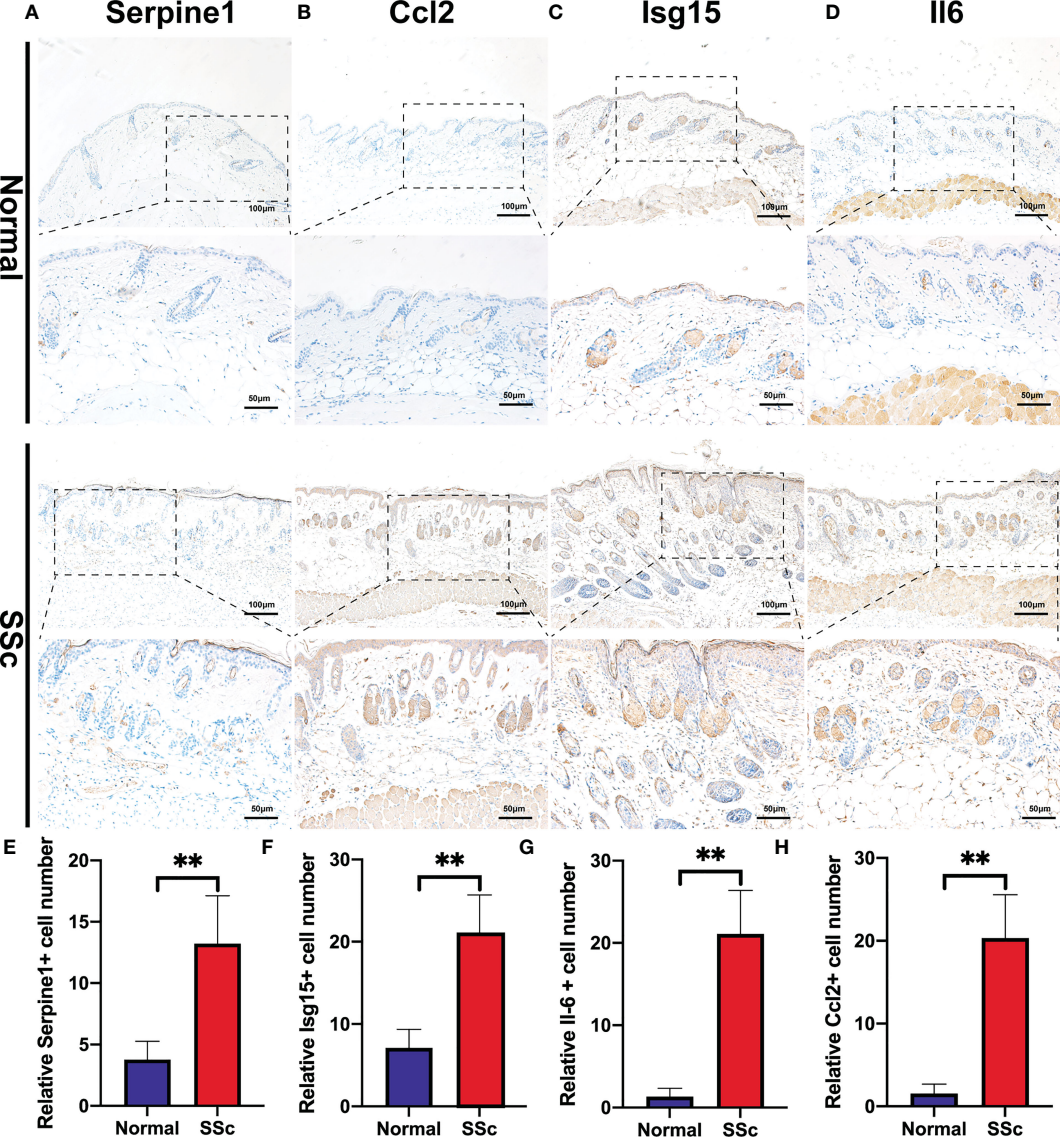
Immunohistochemistry of bleomycin induced scleroderma mouse model and normal control. **(A-D)** Representative images of Serpine1-, Ccl2-, Isg15-, and Il6 -positive cells detected by immunohistochemical staining. **(E-H)** Quantitative analysis of Serpine1-, Ccl2-, Isg15- and Il6 -positive cell numbers. **P < 0.01.

We further confirmed these findings in the SSc mouse model. These revealed four hub genes and proteins were found to be more abundant in the SSc mouse model’s mRNA (**Figures 4K-N**) and protein levels (**Figures 4F-J**) than in normal samples. In addition, the main ECM molecule, including collagen type 1 (Col1a2) and collagen type 3 (Col3a1), is enhanced in the SSc group (**Figures 4O-Q**). It is believed that the IL6-STAT3 signaling pathway integrates multiple profibrotic signals and is a crucial activation checkpoint for fibroblasts (31). We investigated the activation of phospho-STAT3 and STAT3 in the SSc model. As shown in **Figures 4O, R**, phospho-STAT3 was highly increased in SSc skin samples compared to normal samples, suggesting that the STAT3 signaling pathway was activated in SSc. Moreover, immunohistochemical analysis also revealed that the number of Pai-1-, CCL2-, IL6-, and ISG15- positive cells was much higher in the SSc mouse model group than in normal control (**Figures 5A-H**). Thus, SERPINE1, CCL2, IL6, and ISG15 expression was generally higher in the mouse SSc model than in normal samples, and should be the biomarkers of SSc.

### 3.6 ROC curve of the tissue-specific expressed hub genes in SSc

**Figure 6:**
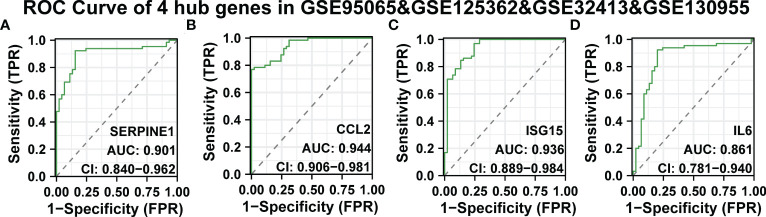
ROC curve of the four specifically expressed hub genes. **(A-D)** The GSE95065, GSE125362, GSE32413, and GSE130955 datasets were used to validate the diagnostic effectiveness of the four specifically expressed hub genes by ROC analysis. AUC is the area under the ROC curve.

Continuously, we evaluated the diagnostic effectiveness of the above SSc tissue-specific expressed hub genes. GSE95065, GSE125362, and GSE32413 were used as test sets, and GSE130955 was used as a validation set. In the GSE95065, GSE125362, and GSE32413, the AUC values of hub genes were as follows: SERPINE1 (AUC: 0.818), CCL2 (AUC: 0.941), ISG15 (AUC: 0.783), and IL6 (AUC: 0.965) (**Supplementary Figures 5A-D**). In the GSE130955 dataset, the AUC values of hub genes were as follows: SERPINE1 (AUC: 0.995), CCL2 (AUC: 0.95), ISG15 (AUC: 0.935), and IL6 (AUC: 0.953) (**Supplementary Figures 5E-H**). Furthermore, we merged four gene matrices to examine the diagnostic value of four hub genes. In the GSE95065, GSE125362, GSE32413, and GSE130955, the AUC values of hub genes were as follows: SERPINE1 (AUC: 0.901), CCL2 (AUC: 0.944), ISG15 (AUC: 0.936), and IL6 (AUC: 0.861) (**Figures 6A-D**). Among them, SERPINE1 has the best diagnostic performance (AUC: 0.944) in SSc.

Due to their strong diagnostic performance in SSc, we combined their expression levels to find better biomarkers. In the SSc group, there was statistically significant up-regulation of SERPINE1, CCL2, IL6, and ISG15 expression levels. According to the analyses, SERPINE1, CCL2, IL6, and ISG15 may be useful biomarkers for SSc.

### 3.7 Construction of the ceRNA networks and the potential RNA regulatory pathways

**Figure 7:**
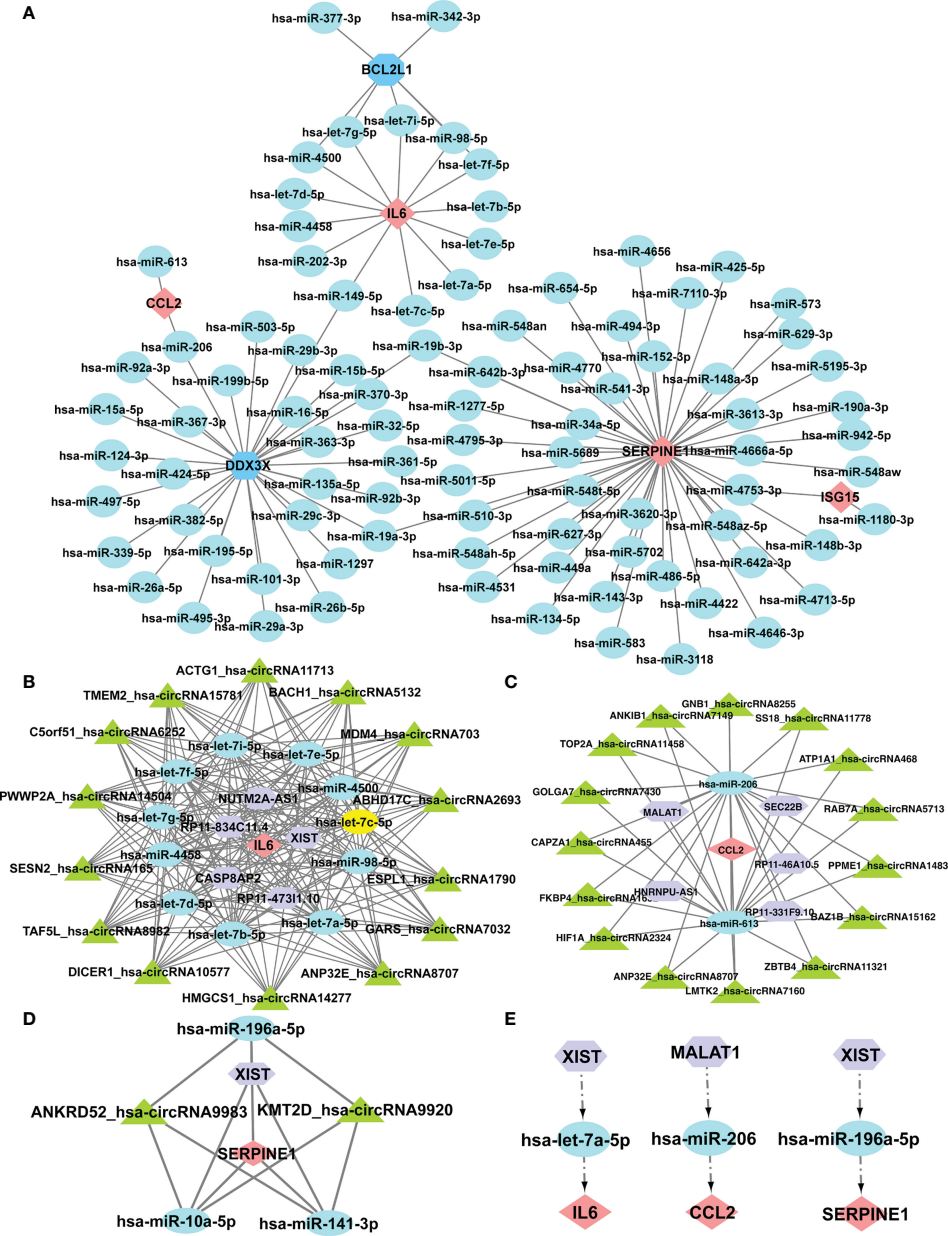
Construction of the ceRNA networks of SERPINE1, CCL2, and IL6 and the potential RNA regulatory pathways. **(A)** Cytoscape created the mRNA-miRNA co-expressed network. SERPINE1 has the most target miRNAs. One node represents an mRNA or miRNA, while one edge represents one interaction between mRNA and miRNA. Pink diamonds and blue octagons represent the hub genes, and blue circles represent miRNAs. The prognostic circRNA-lncRNA-miRNA-mRNA ceRNA network was constructed, including three subnetworks. **(B)** ceRNA network of IL6. **(C)** ceRNA network of CCl2. **(D)** ceRNA network of SERPINE1. **(E)** XIST1-let-7a-5p-IL6, MALAT1-miR-206-CCL2, and XIST1-miR-196a-5p-SERPINE1. Pink diamonds represent the hub genes; blue circles represent miRNAs; a purple octagon represents lncRNAs; and a green triangle represents circRNAs.

It was reported that miRNAs attach to mRNAs and then cause gene silence and downregulate gene expression. Next, five available miRNA databases were applied to estimate the target miRNAs that bind to hub genes. We identified 101 mRNA-miRNA pairings and 92 target miRNAs. Eventually, a miRNA-mRNA network was created using Cytoscape based on the prediction results (**Figure 7A**). These various miRNAs and mRNAs might be crucial nodes in the incidence and progression of SSc. circRNAs and lncRNAs, upstream molecules of miRNA, can influence miRNA’s biological effects and upregulate gene expression. Starbase predicted the lncRNAs and circRNAs that are connected to the selected miRNAs. The criteria for screening were as follows: mammalian, human h19 genome, strict stringency (> = 5) of CLIP-Data, and with or without degradome data. Finally, we selected lncRNAs and circRNAs that are present in the majority of the miRNA prediction findings. The circRNAs with the most samples were screened as the target circRNAs in the circBase database. As shown in **Figures 7B-D**, three ceRNA networks containing hub genes were constructed: 5 lncRNAs and 14 circRNAs of the target miRNAs of IL6; 5 lncRNAs and 15 circRNAs of the target miRNAs of CCL2; and 1 lncRNA and 2 circRNAs of the target miRNAs of SERPINE1. Next, we searched the related literature in accordance with the ceRNA hypothesis. Three downregulated miRNAs and two upregulated lncRNAs were selected for further investigation. The important RNA regulatory pathways, including XIST1-let-7a-5p-IL6 and MALAT1-miR-206-CCL2 and XIST1-miR-196a-5p-SERPINE1 may offer insight into the molecular mechanisms of SSc (**Figure 7E**).

### 3.8 Validation of potential RNA regulatory pathways in the SSc mouse model

**Figure 8:**
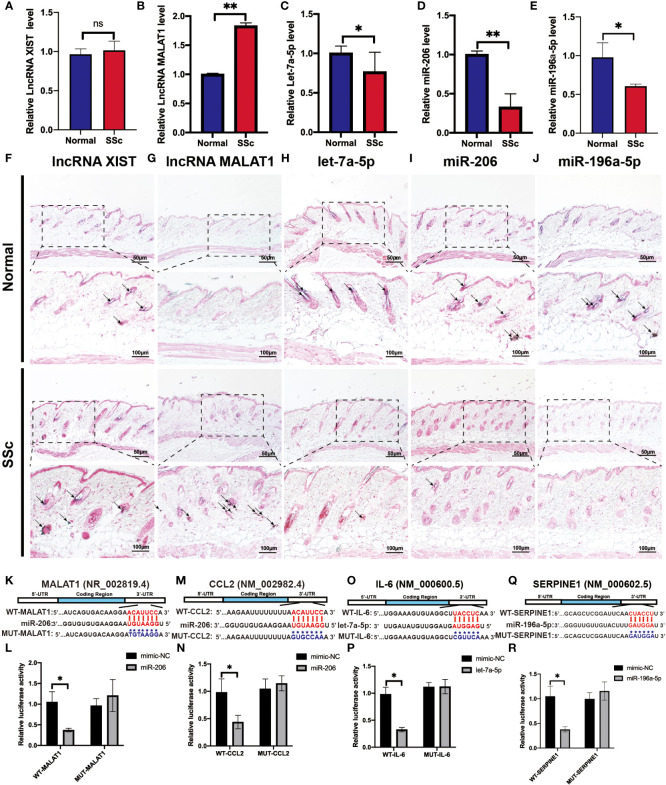
Verification of the potential RNA regulatory pathways in the SSc mouse model. **(A-E)** Quantification of LncRNA XIST, LncRNA MALAT1, let-7a-5p, miR-206, and miR-196a-5p mRNA in normal control mice and the SSc mouse model. **(F-J)** Representative ISH images showed the expression of LncRNA XIST, LncRNA MALAT1, let-7a-5p, miR-206, and miR-196a-5p in normal control mice and the SSc mouse model. **(K)** Potential binding sites of MALAT1 to miR-206. **(L)** Luciferase reporter gene assays were performed to confirm the direct targeting of MALAT1 to miR-206. **(M)** Potential binding sites of CCL2 to miR-206. **(N)** Luciferase assays were performed to confirm the direct targeting of CCL2 to miR-206. **(O)** Potential binding sites of IL-6 to let-7a-5p. **(P)** Luciferase reporter gene assays were performed to confirm the direct targeting of IL-6 to let-7a-5p. **(Q)** Potential binding sites of SERPINE1 to miR-196a-5p. **(R)** Luciferase reporter gene assays were performed to confirm the direct targeting of SERPINE1 to miR-196a-5p. Scale bar 50 and 100 μm. *P < 0.05, **P < 0.01 ns, means no significant differences. Black arrows represent positive in situ hybridization signals (blue-purple).

To validate the above analyzed RNA regulatory pathways, RT-PCR was used to detect the hub genes of the normal mice and the SSc mouse model firstly. As shown in **Figures 8A-E**, the expression of MALAT1 (P < 0.05) significantly increased in the SSc group compared with normal mice. But let-7a-5p (P < 0.05), miR-206 (P < 0.05), and miR-196a-5p (P < 0.05) decreased in the SSc mouse model. And XIST did not show significant differences. Moreover, we applied ISH to detect the location and expression of these lncRNA and miRNA in the skin of the SSc mouse model. As shown in **Figures 8F-J**, the majority of the positive signals (blue-purple) for MALAT1, XIST, let-7a-5p, miR-196a-5p, and miR-206 were found in the dermal layer. The alteration of these lncRNA and miRNA expression was consistent with the RT-PCR results. According to these results, MALAT1, let-7a-5p, miR-206, and miR-196a-5p are more promising candidates for further investigation.

Next, we applied the luciferase assay to investigate and confirm the substantial interaction between these genes. As shown in **Figures 8H-P**, miR-206-MALAT/CCL2, let-7a-5p-IL-6 and miR-196a-5p-SERPINE1 had significantly lower luciferase activity compared to the ones containing mimic-NCs (P < 0.05). And there is no significant difference in the luciferase activity was observed in cells transfected with a mutant binding sequence. These results revealed that the interaction of miR-206 to MALAT and CCL2, let-7a-5p to IL-6, and miR-196a-5p to SERPINE1 may play an essential role in regulating the progression of SSc.

### 3.9 Drug-gene interaction analyses screen prospective medicines

**Figure 9:**
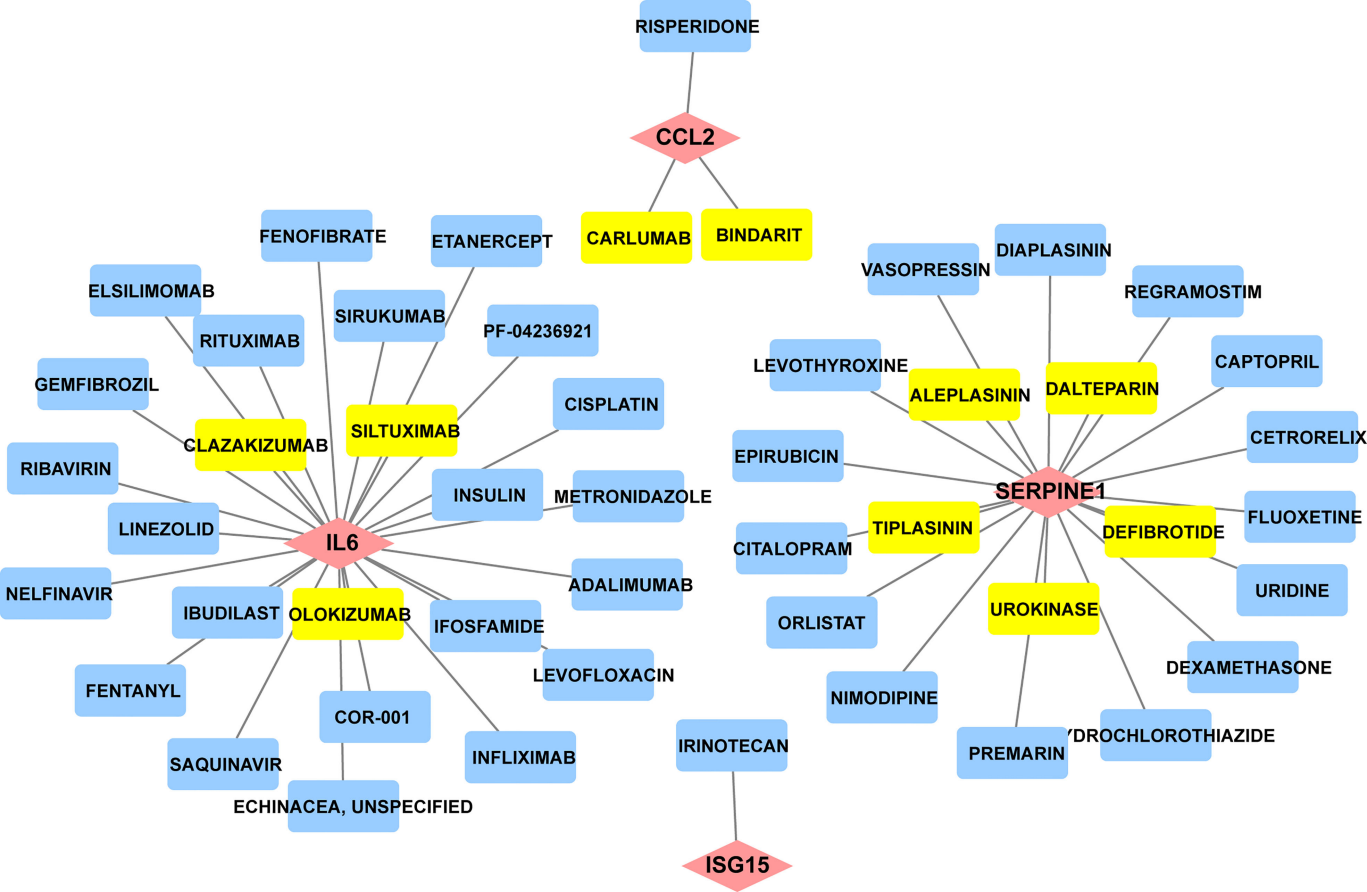
Constructed the network of drug-gene interactions. The pink diamonds represent the hub genes, yellow round rectangle represent the top ten drugs with the highest scores, blue round rectangle represent other potential drugs. The scores of potential drugs identified by the DGIdb show the importance of drugs, with a higher score indicating greater importance.

Since there remain unmet medical needs, in-depth studies on the potential drug for scleroderma are necessary. SERPINE1, CCL2, IL6, and ISG15 were identified as effective biomarkers and possible drug targets for SSc. To further identify potential drugs for SSc, the above 4 biomarkers were analyzed in the DGIbd database. A total of 49 medications were selected, and Cytoscape was applied to show how the drugs associate with hub genes (**Figure 9**). SERPINE1 (36.36, 8/22), CCL2 (9.09%, 2/22), ISG15 (4.55%, 1/22), and IL6 (50.00%, 11/22) genes are attractive candidates for future anti-skin fibrosis medications. The information of top 10 drugs (CARLUMAB, BINDARIT, OLOKIZUMAB, SILTUXIMAB, CLAZAKIZUMAB, TIPLASININ, DEFIBROTIDE, UROKINASE, ALEPLASININ, and DALTEPARIN) was shown in **Table 4**. CARLUMAB and BINDARIT ranked the top two in interaction score. In addition, among five SERPINE1 inhibitors, ALEPLASININ has the highest interaction score. Therefore, CARLUMAB, BINDARIT and ALEPLASININ might be potential drugs for SSc.

**Table 4:** Top ten drugs screened in DGIdb based on score ranking.

Drugs	Interaction Type & Directionality	Interaction score	Target Gene
CARLUMAB	inhibitor (inhibitory)	41.22	CCL2
BINDARIT	n/a	20.61	CCL2
OLOKIZUMAB	inhibitor (inhibitory)	9.89	IL6
SILTUXIMAB	antagonist (inhibitory)	9.89	IL6
CLAZAKIZUMAB	inhibitor (inhibitory)	7.42	IL6
TIPLASININ	n/a	6.18	SERPINE1
DEFIBROTIDE	n/a	3.09	SERPINE1
UROKINASE	inducer (activating), substrate	3.09	SERPINE1
ALEPLASININ	inhibitor (inhibitory)	3.09	SERPINE1
DALTEPARIN	n/a	3.09	SERPINE1

n/a means not applicable.

## 4 Discussion

Systemic sclerosis (SSc) is a complex autoimmune disease characterized by skin inflammation, vasculopathy, and excessive fibrosis in multiple organs. There are no ideal therapies at this time due to the complexity and variety of SSc. Therefore, recognizing potential biomarkers, exploring the precise pathogenesis of SSc and finding therapeutic targets are significant for current research. To date, the mouse SSc model was induced by daily local injections of bleomycin (BLM) solution and was widely applied to study the treatment and investigation of SSc. The sites of the skin distant from the sites of injection failed to show any sclerosis. So, the effects of BLM injection on the development of sclerotic skin lesions are local, rather than systemic in mice (22). However, the injection site of the SSc mouse is similar to the histologic features of human scleroderma skin, which show progressive skin thickening, excessive accumulation of collagen, immune cell infiltration, and thickening of the vascular wall, as well as to the cell biological characteristics, such as the fact that the number of mast cells grew steadily as the sclerotic alterations progressed, anti-nuclear antibodies were also detected in the serum of BLM-treated mice, and TGFβ1 mRNA was discovered at an early stage (32, 33). Thus, we confirmed tissue-specifically expressed hub genes and the potential RNA regulatory pathways in this mouse model.

Here we identified 254 DEGs and found that the haematologic/immune system and bone/muscle tissue have the highest distribution of DEGs of SSc. The DEGs concentrated on metabolism, regulation of actin cytoskeleton, VEGF signaling pathway, and immune-related processes, which is important in the SSc processes. And we identified 4 hub genes, including Serpine1, CCL2, IL6, and ISG15. The mRNA and protein expression of hub genes was greatly increased in SSc patients and bleomycin induced mouse model. Combined with ROC curve analysis, we consider SERPINE1, IL6, CCL2, and ISG15 may be novel and potential biomarkers for SSc.

SERPINE1, also known as Plasminogen activator inhibitor (PAI-1), is the main physiologic inhibitor of the plasmin-based pericellular cascade and prevents the breakdown of the ECM to inhibit the progression of fibroproliferative diseases (34, 35). SERPINE1 plays a crucial role in the TGF-1/SMAD3-induced pathway, which is related with tissue fibrosis, including SSc (36–38). The five potential drugs for SSc, both TIPLASININ, DEFIBROTIDE, UROKINASE, ALEPLASININ, and DALTEPARIN, are inhibitors of PAI-1 and block the blood clotting cascade. Among them, ALEPLASININ has the highest interaction score. TIPLASININ (synonyms: PAI-039; TIPLASININ) is reported as an orally bioavailable antagonist of PAI-1. Tessa et al. indicated that TIPLAXTININ treatment inhibited the recruitment and differentiation of fibroblasts in wounds, which showed the significance of PAI-1 inhibitors (39). In conjunction with our analysis, SERPINE1 may be inextricably related to the development of SSc, as well as a useful biomarker for determining which patients should be considered for aggressive therapy and/or clinical trials (40).

For CCL2, IL-6 and ISG15, an increasing body of literature has revealed that the three biomarkers are strongly related to inflammation, but as previously stated, there are indeed tough questions that need to be answered regarding the detailed mechanism and relationship to SSc. CCL2 promotes the migration and activation of monocytes and T cells (41, 42),and is highly expressed in serum or bronchoalveolar lavage (BAL) fluids in SSc (43). IL-6 mainly released by T cells, monocytes, and fibroblasts (44), is enhanced in the serum of diffuse cutaneous systemic sclerosis (dcSSc) and strongly related to skin thickness (45). Consistent with these findings, our study revealed that CCL2 and IL-6 was considerably elevated in the skin of SSc samples and was highly diagnostic for SSc. ISG15 is a type I IFN inducible gene that acts as a negative regulator of type I IFN induction (46). Patients with SSc exhibit dysregulation of the type I IFN pathway at the epigenetic level, suggesting that hypomethylation and overexpression of type I IFN-related genes may be crucial in SSc pathogenesis (47). CARLUMAB and BINDARIT are CCL2 inhibitors in our prospective medicine screening and rank the top two in interaction score. CARLUMAB was well-tolerated in metastatic castration-resistant prostate cancer (48). Moreover, a clinical trial indicated that CARLUMAB treatment did not provide benefit in patients with idiopathic pulmonary fibrosis (49). BINDARIT treatment may alleviate subsynovial connective tissues fibrosis in Carpal Tunnel Syndrome CTS and improved vascular dysfunction and lung inflammation in radiation-induced lung injury (50, 51). These finding suggests that the therapeutic role of CARLUMAB and BINDARIT is also worth investigating in SSc.

The ceRNA activity expands the functional genetic data in the human genome and is crucial in pathological circumstances (52). We considered that XIST1-miR-196a-5p-SERPINE1, XIST1-let-7a-5p-IL6, and MALAT1-miR-206-CCL2 may be crucial in the progression of SSc. It was discovered that the level of lncRNA MALAT1 in white blood cells (WBCs) was upregulated in the SSc compared to the controls. miRNA microarray chip analysis also identified the downregulation of hsa-miR-206 in the skin samples of SSc (53). In our study, RT-PCR, luciferase experiment, and ISH supported the MALAT1-miR-206-CCL2 ceRNA interaction. Furthermore, lncRNAs XIST can regulate other epigenetic processes and increased in other autoimmune diseases such as multiple sclerosis (MS) (54) and systemic lupus erythematosus (SLE) (55). Let-7a expression was lower in SSc and LSc skin than in normal skin, both *in vivo* and *in vitro* (56). It reported that real-time PCR and *in situ* hybridization revealed that the expression of miR-196a was lower in the skin of SSc patients than in controls (57). And luciferase assay demonstrated that SERPINE1 is a direct target of miR-196a-5p, and IL6 is a direct of let-7a-5p. For the diagnosis and treatment of SSc, a further evaluation of miR-196a-5p-SERPINE1, let-7a-5p-IL6, and MALAT1-miR-206-CCL2 should be beneficial.

Using bioinformatics techniques, our study constructed a ceRNA network, analyzed the primary functions of hub genes, and predicted innovative and effective medications, therefore shedding new light on SSc pathogenesis. We anticipate that the present findings will highlight the importance of combining PAI-1 and inflammatory factors in the therapy and diagnosis of SSc. However, the size of patient samples is very modest. Consequently, future research must increase the sample size to further validate our conclusion.

## 5 Conclusion

This study revealed tissue-specific expressed genes, SERPINE1, CCL2, IL6, and ISG15, as effective biomarkers for SSc and provided a certain point of reference for the mechanisms at the transcriptome level. Additionally, we developed the ceRNA network and proposed that the potential RNA regulatory pathways XIST1-miR-196a-5p-SERPINE1, XIST1-let-7a-5p-IL6, and MALAT1-miR-206-CCL2 may be responsible for controlling the progression of SSc. Moreover, PAI-1 and inflammatory factors may be possible drug targets and the drug-hub gene interaction analyses predicted TIPLASININ, CARLUMAB and BINDARIT as candidate drugs for SSc.

## Data availability statement

The original contributions presented in the study are included in the article/**Supplementary Material**. Further inquiries can be directed to the corresponding authors.

## Ethics statement

The animal study was reviewed and approved by Committee on the Ethics of Laboratory Animal Resources, Tongji University (TJAA09622102).

## Author contributions

JJ designed the experiments, analyzed the data, and drafted the paper. MJ and JC analyzed the data and drafted the paper. XY, YL, QO and XZ analyzed the data. CJ and QO contributed to designing the experiment, manuscript drafting and analysis tools. JZ contributed to overall supervising the project, designing the experiment, manuscript drafting and revising as well as final approval of manuscript submission. All authors have agreed on the journal to which the article will be submitted. All authors agree to take responsibility and be accountable for the contents of the article.
