# Supplementary material for: Bioinformatics-integrated screening of systemic sclerosis-specific expressed markers to identify therapeutic targets
Source: Front Immunol. 2023 Mar 30;14:1125183. doi: 10.3389/fimmu.2023.1125183 (PMC10098096; doi:10.3389/fimmu.2023.1125183)
Supplement: Supplementary file 1 [file DataSheet_1.docx]

Supplementary Material

# Supplementary Figures and Tables

## Supplementary Figures

**
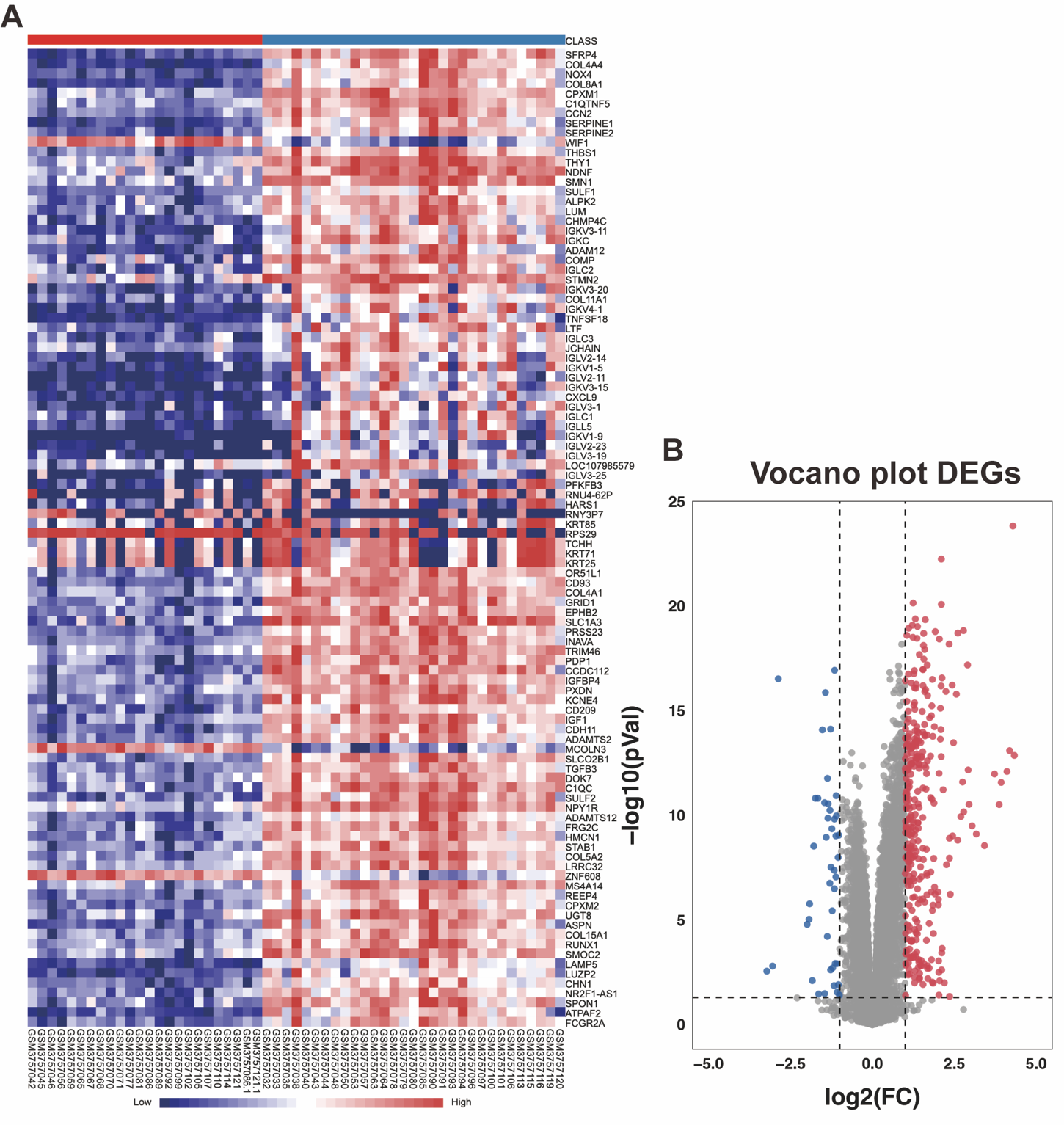
Supplementary Figure 1.** Identification of differentially expressed mRNAs (DEGs) in GSE130955. **E** Heatmap of DEGs between the SSc group and the normal group (red represents high expression; blue represents low expression). **F** Volcano plot of DEGs between the SSc group and the normal group.


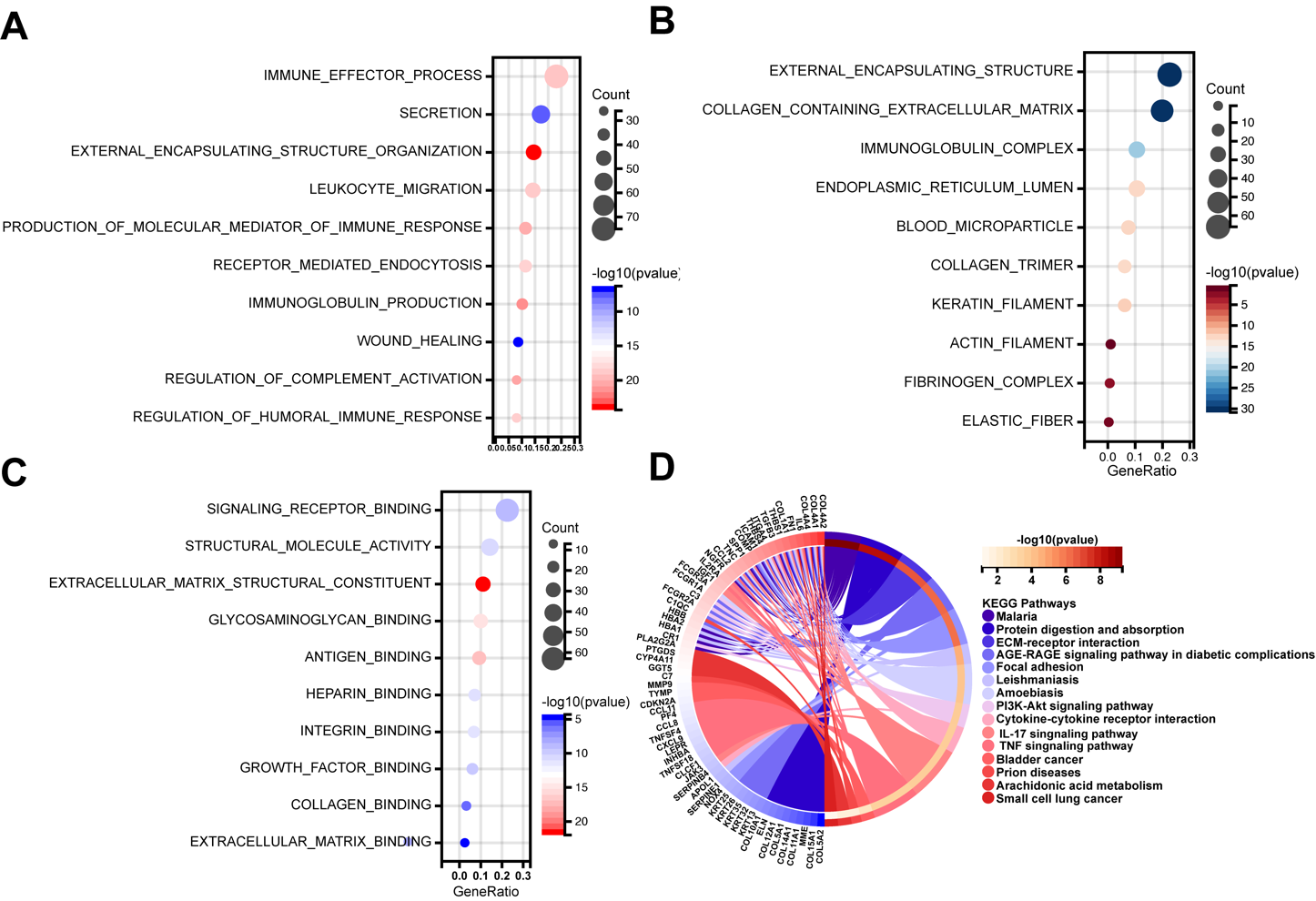
**Supplementary Figure 2.** GO and KEGG pathway enrichment analyses of GSE130955. **A** The bubble plot shows the top 10 enriched biological processes of DEGs. **B** The bubble plot shows the top 10 enriched cellular components of DEGs. **C** The bubble plot shows the top 10 enriched molecular functions of DEGs. **D** The chord plot shows the most enriched KEGG pathways of DEGs.


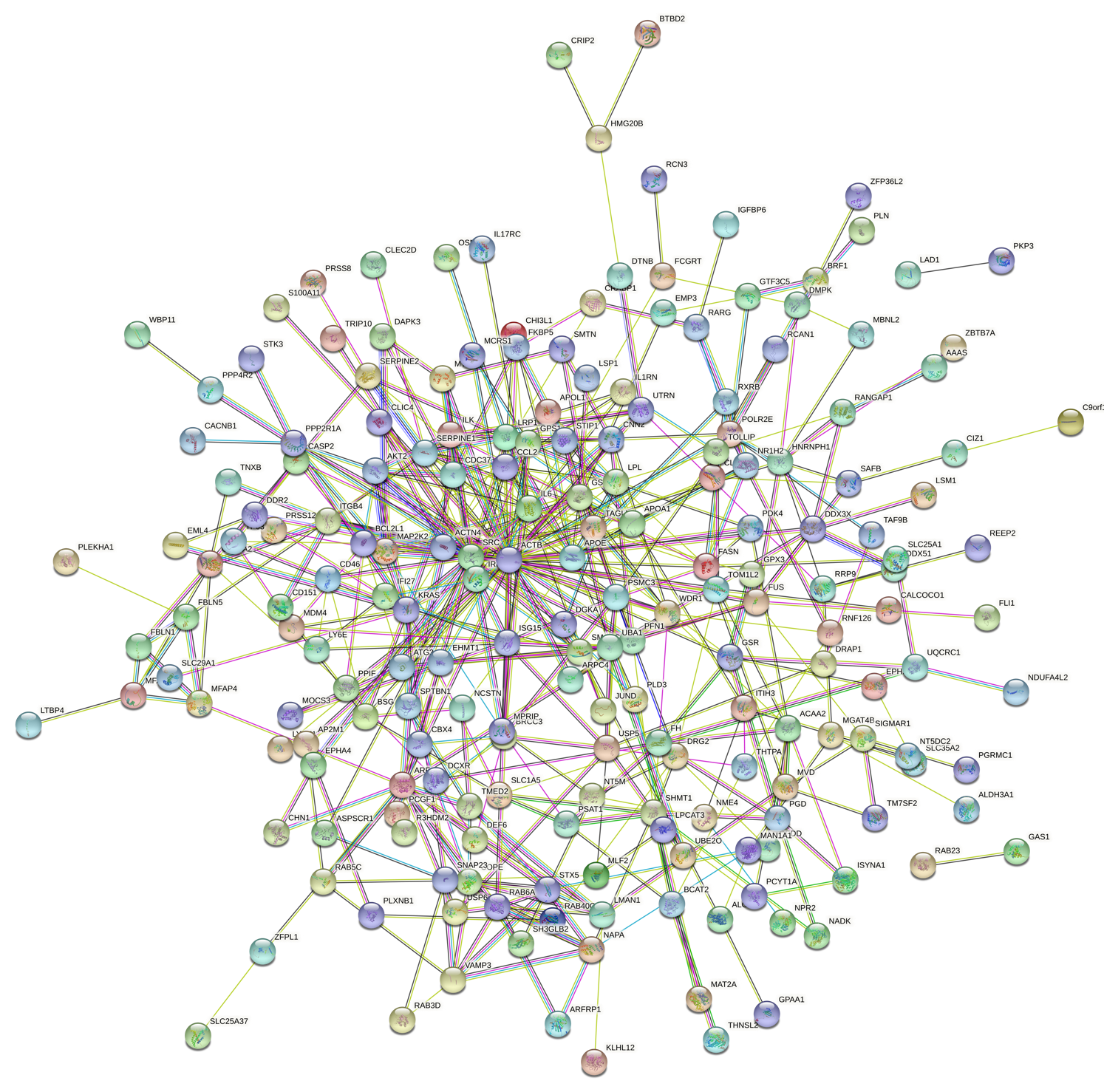
**Supplementary Figure 3.** PPI network results of DEGs by STRING.


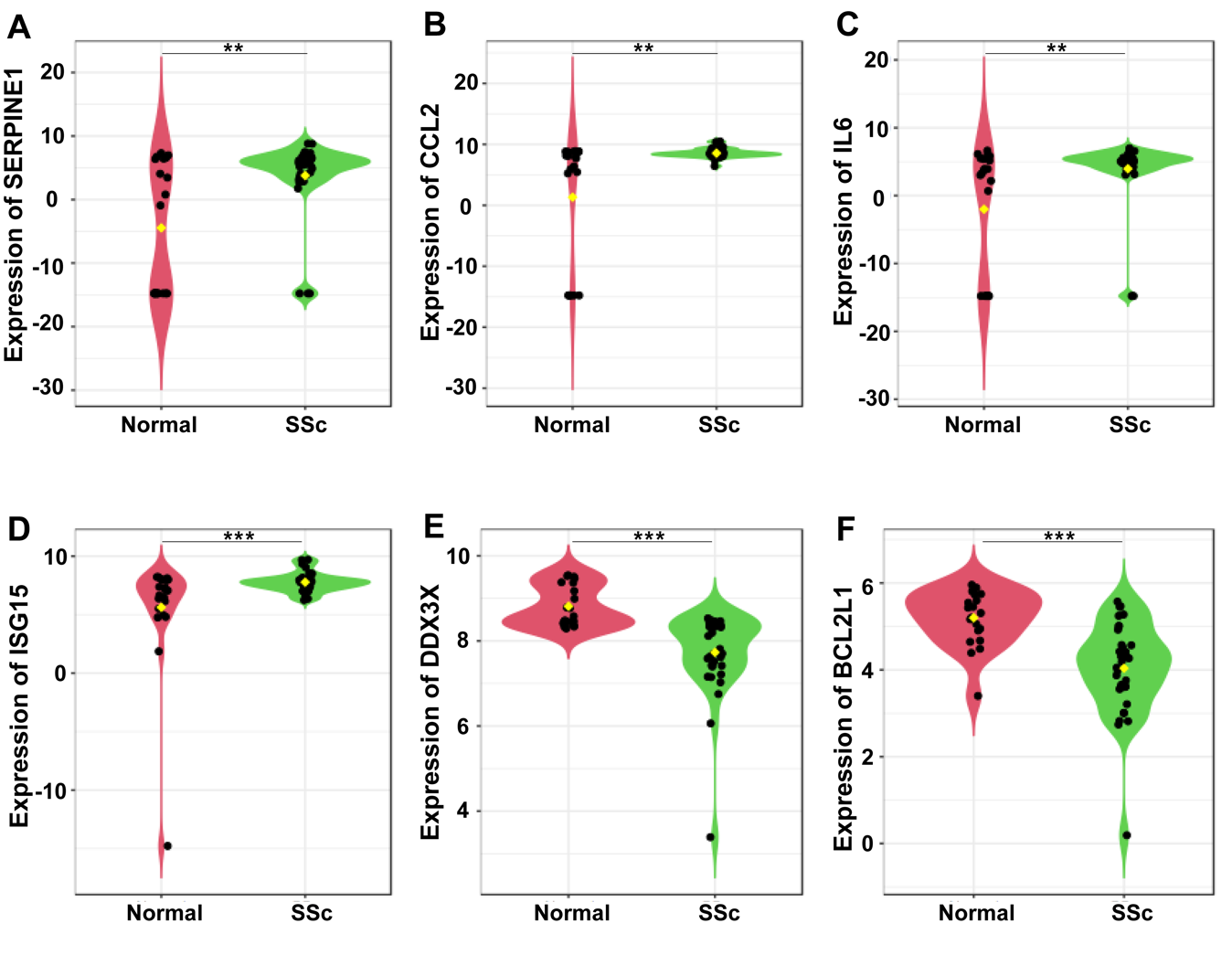


**Supplementary Figure 4** The violin plot showed the detailed expression of four tissue-specifically expressed hub genes using merged microarray data.


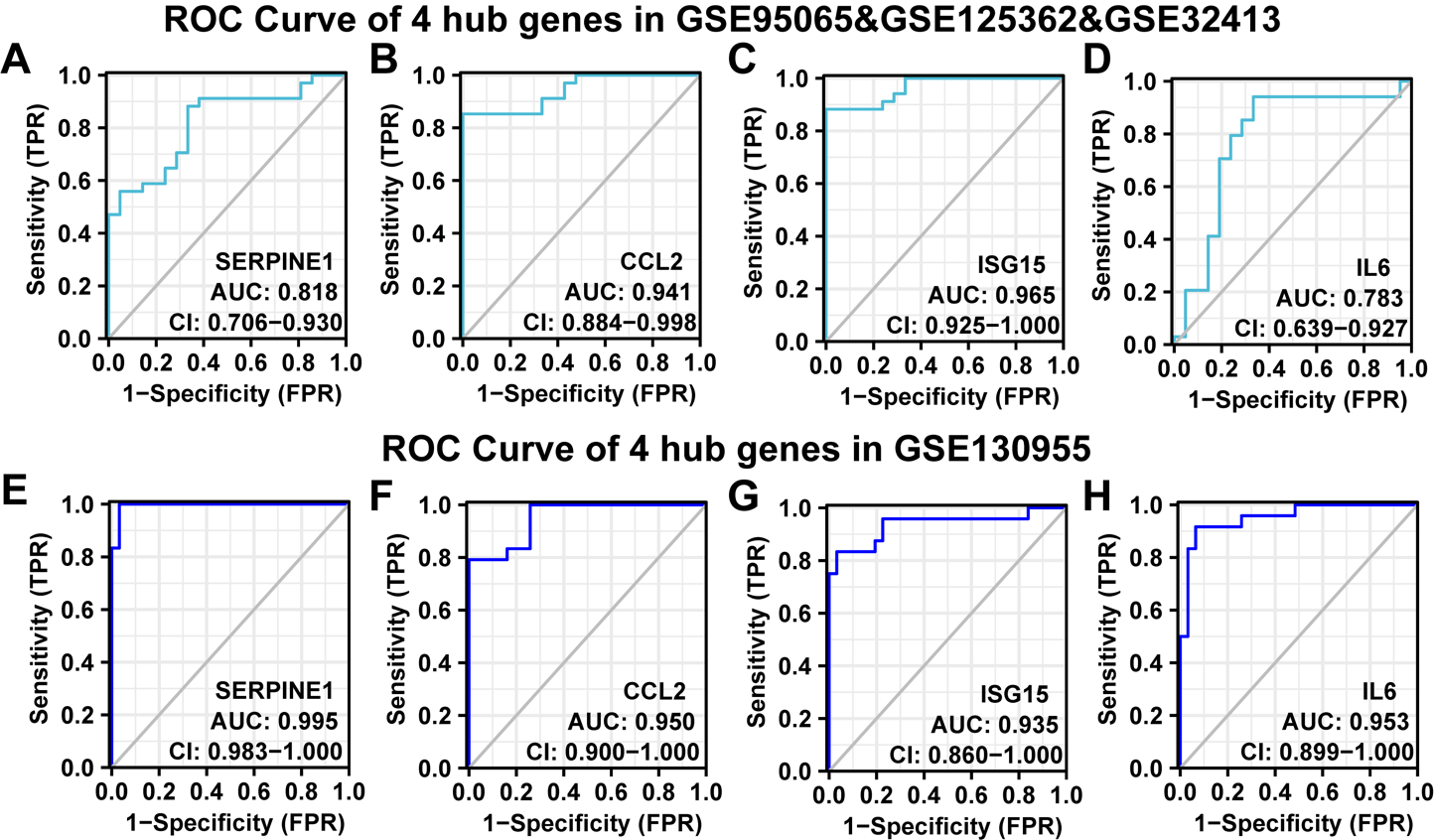


**Supplementary Figure 5** ROC curve of the 4 specifically expressed hub genes. A-D The GSE95065, GSE125362 and GSE32413 dataset was used to validate the diagnostic effectiveness of the 4 specifically expressed hub genes by ROC analysis. E-F The GSE130955 dataset was used to validate the diagnostic effectiveness of the 4 specifically expressed hub genes by ROC analysis. AUC area under the ROC curve.

## Supplementary Table

**Supplementary Table 1:Primers used in RT-PCR experiments**

| Primer | Forward (5’-3’) | Reverse (5’-3’) |
| --- | --- | --- |
| Serpine1 | TGTGGTCTTCTCTCCCTATG | CCCATAGCATCTTGGATCTG |
| Ccl2 | GAATGGGTCCAGACATACATTA | TACGGGTCAACTTCACATTC |
| Isg15 | CAGTGATGCTAGTGGTACAG | GCGTCAGAAAGACCTCATAG |
| Il-6 | GAGGAGACTTCACAGAGGATA | ATTTCCACGATTTCCCAGAG |
| Gapdh | ATGGGGAAGGTGAAGGTCG | GGGGTCATTGATGGCAATA |

**Supplementary Table 2: Antibodies used in immunostaining and WB**

| Antibodies | Cat No. | Brand | Dilution ratio |
| --- | --- | --- | --- |
| Pai-1 | 66261-1-Ig | Proteintech | 1:5000 |
| Ccl-2 | 25542-1-AP | Proteintech | 1:1000 |
| Isg15 | 15981-1-AP | Proteintech | 1:1000 |
| Il6 | 12912 | Cell Signaling Technology | 1:1000 |
| Gapdh | 60004-1-Ig, | Proteintech | 1:5000 |

**Supplementary Table 3: Probe used in ISH experiments**

| Probe | Sequences (5’-3’) |
| --- | --- |
| miR-196a-5p | 5’-ATCCATCAAAGTACAACAACCC-3’Dig |
| miR-206 | 5’-ACCTTACATTCCTTCACACACC-3’Dig |
| Let-7a-5p | 5’-ACTCCATCATCCAACATATCAA-3’Dig |
| LncRNA MALAT | 5’-TGCCTCACGAGCTCAGCTGTGCTGCTCTACGCTGCTCTGCTCTC  GCTGCC-3’Dig |
| LncRNA Xist | 5’AGAAACCACGGAAGAACCGCACATCCACGGGAAACGAGCAAA  CATGGCTG-3’Dig |
